# What Live I for?

**Published:** 1887-08-13

**Authors:** 


### WHAT LIVE I FOR?

I live for those who love me,
For those who know me true;
For the heaven that smiles above me,
And awaits my spirit too;
For the cause that lacks assistance,
For the wrong that needs resistance,
For the future in the distance,
For the good that I can do.

